# Fundamental Body Composition Principles Provide Context for Fat-Free and Skeletal Muscle Loss With GLP-1 RA Treatments

**DOI:** 10.1210/jendso/bvae164

**Published:** 2024-09-25

**Authors:** Grant M Tinsley, Steven B Heymsfield

**Affiliations:** Energy Balance & Body Composition Laboratory, Department of Kinesiology & Sport Management, Texas Tech University, Lubbock, TX 79409, USA; Pennington Biomedical Research Center, Louisiana State University System, Baton Rouge, LA 70808, USA

**Keywords:** obesity, glucagon-like peptide-1 receptor agonist, lean mass, lean body mass, weight loss, exercise

## Abstract

During weight loss, reductions in body mass are commonly described using molecular body components (eg, fat mass and fat-free mass [FFM]) or tissues and organs (eg, adipose tissue and skeletal muscle). While often conflated, distinctions between body components established by different levels of the 5-level model of body composition—which partitions body mass according to the atomic, molecular, cellular, tissue/organ, or whole-body level—are essential to recall when interpreting the composition of weight loss. A contemporary area of clinical and research interest that demonstrates the importance of these concepts is the discussion surrounding body composition changes with glucagon-like peptide-1 receptor agonists (GLP-1RA), particularly in regard to changes in FFM and skeletal muscle mass. The present article emphasizes the importance of fundamental principles when interpreting body composition changes experienced during weight loss, with a particular focus on GLP-1RA drug trials. The potential for obligatory loss of FFM due to reductions in adipose tissue mass and distribution of FFM loss from distinct body tissues are also discussed. Finally, selected countermeasures to combat loss of FFM and skeletal muscle, namely resistance exercise training and increased protein intake, are presented. Collectively, these considerations may allow for enhanced clarity when conceptualizing, discussing, and seeking to influence body composition changes experienced during weight loss.

Obesity treatment continues to garner substantial attention due to the notable prevalence and increasing rates of obesity worldwide [1], as well as the promise of emerging treatment options [2]. While a shift in the focus of obesity treatment from weight management alone to improving patient-centered health outcomes has been promoted [3], weight loss remains a typical treatment goal. In most cases, weight loss is composed of losses both in fat mass (FM) and fat-free mass (FFM). A frequent goal of weight loss therapies is to maximize the percentage of weight lost as FM, thereby minimizing loss of FFM. The reasoning provided for this goal is often the preservation of skeletal muscle mass due to its fundamental metabolic and functional importance [4, 5]. However, while often conflated, FFM and skeletal muscle mass are distinct entities with overlap in the molecules they comprise. Additionally, inconsistent use of terms related to FFM—such as *lean mass*, *lean body mass*, and *lean soft tissue*—may add to the confusion when discussing body composition changes with weight loss. This is not a new concern, as this specific terminological issue was highlighted more than 3 decades ago [6]. Prior investigations, including some discussed in the present article, provide ambiguous descriptions of “lean mass,” creating confusion regarding which specific component is being discussed (eg, FFM vs lean soft tissue). Here, we preferentially use the term *FFM*, with “lean” used periodically as a more general reference to either nonfat or nonadipose tissue components (Table 1 provides recommended terminology).

**Table 1.** Recommended body composition terminology

Term(s)	Definition	Body composition level	Common measurement techniques	Notes
Fat-free mass Lean body mass^a^	The estimated mass of all nonfat molecules in the body, regardless of where they occur. In this case, “fat” refers to nonpolar lipids, mainly triglycerides	Molecular	DXA, BIA, BIS, ADP, UWW, 3DO, SKF, anthropometric equations	To avoid confusion relative to the historical use of “lean body mass,” *fat-free mass* is the preferred term for this body component
Lean mass	A general term to refer to nonfat or nonadipose tissue components	Not specified	N/A	This term may be used in general discussions as a synonym of FFM or adipose tissue-free mass but lacks a clear singular definition as a body component.
Lean soft tissue mass	The estimated mass of all nonfat, non-bone mineral molecules in the body, regardless of where they occur. In this case, “fat” refers to nonpolar lipids, mainly triglycerides	Molecular	DXA	While DXA can estimate lean soft tissue due to its estimation of bone mineral mass, other techniques (eg, BIA) may report “lean soft tissue” as an outcome when equations are calibrated to DXA lean soft tissue
Fat mass	The estimated mass of all fat molecules in the body, regardless of where they occur. In this case, “fat” refers to nonpolar lipids, mainly triglycerides	Molecular	DXA, BIA, BIS, ADP, UWW, 3DO, SKF, anthropometric equations	Historically, there has been debate regarding the presence or absence of “essential fat” (ie, structural lipids such as phospholipids and sphingomyelin) within this component. Here, “fat mass” is recommended to refer only to nonpolar lipids, mainly triglycerides
Adipose tissue mass	The estimated mass of all anatomically defined adipose tissue in the body	Organ/Tissue	MRI, CT, US (local assessments)	This body component should only be reported by appropriate imaging techniques or methods employing sufficiently validated equations based on such techniques
Skeletal muscle mass	The estimated mass of all anatomically defined skeletal muscles in the body	Organ/Tissue	MRI, CT, US (local assessments)	This body component should only be reported by appropriate imaging techniques or methods employing sufficiently validated equations based on such techniques

Abbreviations: 3DO, 3-dimensional optical imaging; ADP, air displacement plethysmography; BIA, bioelectrical impedance analysis; BIS, bioimpedance spectroscopy; CT, computed tomography; DXA, dual-energy x-ray absorptiometry; FFM, fat-free mass; MRI, magnetic resonance imaging; SKF, skinfold thickness assessments; US, ultrasonography; UWW, underwater weighing.

^*a*^Historical definitions of lean body mass have been debated. For scientific and pragmatic reasons, we believe that the terms “*fat-free mass*” and “*lean body mass*” are best viewed as equivalent.

The concern regarding lean and skeletal muscle loss during weight loss has been the subject of recent scientific and general discussion due to contentions that glucagon-like peptide-1 receptor agonist (GLP-1RA) drugs may cause disproportionate loss of these components [7-9]. To better inform these conversations and promote accurate interpretation of contemporary clinical trials, a recollection of established body composition principles is needed. As such, the purpose of this article is to demonstrate the importance of fundamental body composition concepts for interpreting FFM and skeletal muscle changes experienced during weight loss, with a particular focus on GLP-1RA drugs. Additionally, selected countermeasures to combat loss of FFM and skeletal muscle, namely resistance training and increased protein intake, are discussed. For this article, the historical and contemporary body composition literature was searched for relevant research establishing fundamental principles or including illustrative body composition changes.

## The 5-Level Model

**Figure 1.**
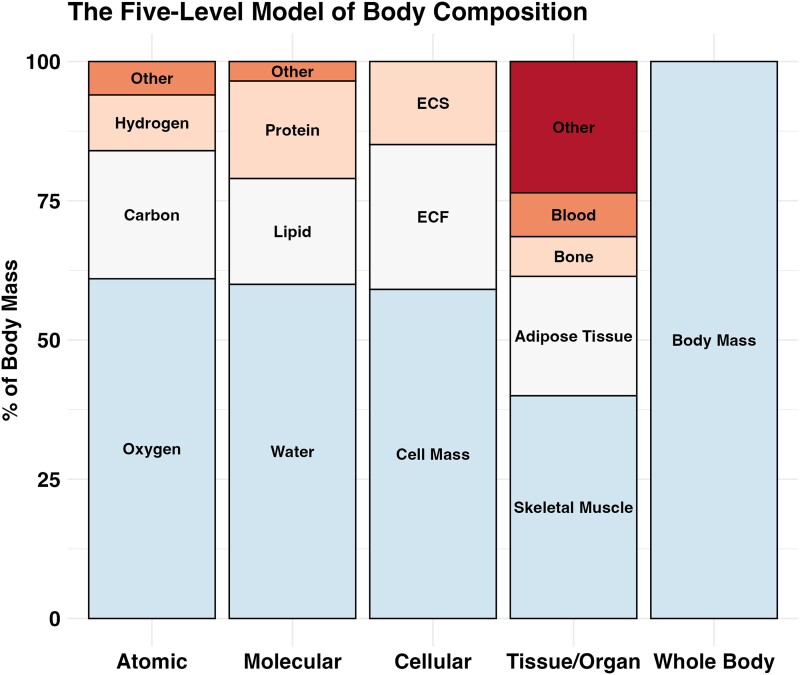
The 5-level model of body composition. Adapted with permission from Wang et al [6]. Proportions are for illustrative purposes and are based on values from the *Report of the Task Group on Reference Man* [10]. Abbreviations: ECF, extracellular fluid; ECS, extracellular solids.

**Figure 2.**
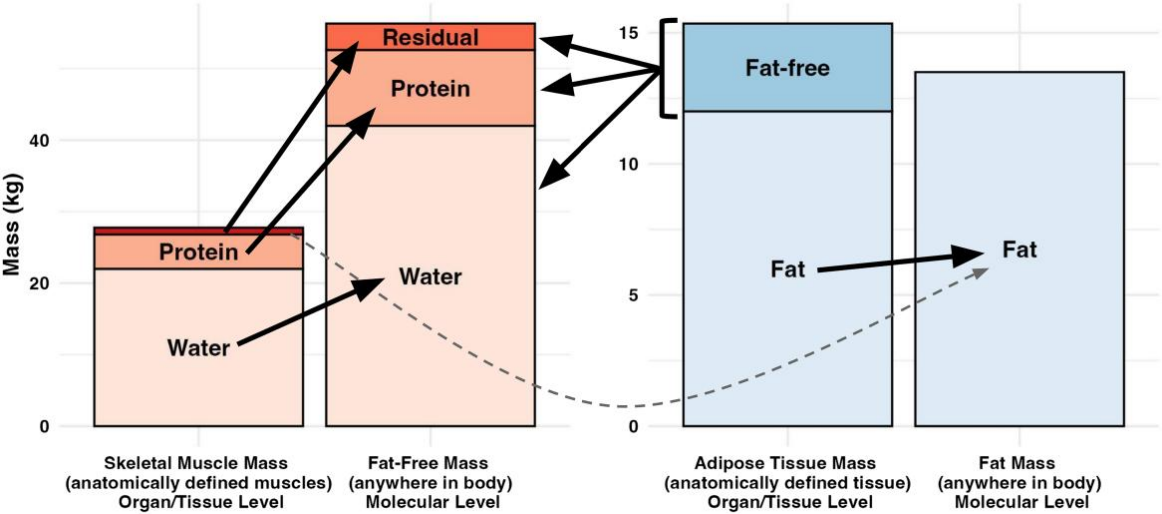
Interrelationships between molecular and organ/tissue levels of body composition assessment. Solid arrows indicate the molecules within anatomically defined organ/tissue components (ie, skeletal muscle and adipose tissue) contributing to molecular-level components (ie, fat-free mass and fat mass). “Fat” refers to nonpolar lipids, mainly triglycerides. In contrast, structural or polar lipids (eg, phospholipids, sphingomyelin), historically termed “essential fat,” are included within fat-free components. The dashed line indicates the small contribution of skeletal muscle triglyceride to fat mass. The small unlabeled portion at the top of the skeletal muscle mass stacked bar corresponds to the residual content of skeletal muscle (ie, fat, mineral, structural lipids, glycogen, and other small components). The bracketed portion of the adipose tissue mass stacked bar corresponds to the fat-free (lean) component of adipose tissue, containing water, protein, mineral, structural lipids, glycogen, and other small components. Data are for illustrative purposes and are based on values from the *Report of the Task Group on Reference Man* [10].

In their seminal 1992 publication, Wang et al [6] defined the 5-level model of body composition to promote a more comprehensive and accurate system of body composition organization. This model allows for the categorization of total body mass according to 5 distinct levels: atomic, molecular, cellular, tissue/organ, and whole body (Fig. 1). Today, most accessible body composition assessment methods are based on the molecular level, which provides common metrics, such as body fat percentage, FM, and FFM. While familiar and useful, these metrics do not respect anatomy and simply represent the cumulative masses of different molecular components, wherever they occur in the body. In contrast, clinicians and researchers are often interested in understanding the quantities of anatomically defined components with physiological relevance, such as adipose tissue and skeletal muscle. As such, FM is often conflated with adipose tissue, and FFM is often conflated with skeletal muscle. However, these seemingly analogous pairs are in fact distinct in several regards. First, as stated, they belong to different levels of assessment according to the 5-level model, meaning that they are conceptually distinct. Related to this, the anatomical location of adipose tissue can be defined and investigated through imaging or dissection, while FM is distributed throughout the body, albeit with much of molecular fat typically occurring within adipose tissue. Despite this overlap, the complete molecular makeup of these paired entities differs (Fig. 2). FM is, by definition, entirely composed of fat molecules (ie, nonpolar lipids, mainly triglycerides), while adipose tissue is predominantly fat molecules (∼80%-85%), with additional contributions from water (∼15%) and protein (∼5%) [10]. When comparing the absolute quantity of FM and adipose tissue, values from reference data are similar (13.5 kg FM vs 15 kg adipose tissue in reference man; 16 kg FM vs 19 kg adipose tissue in reference woman [10]). While the approximately 80% to 85% of fat molecules from adipose tissue are also contained within FM, so are all other fat molecules distributed throughout the rest of the body. Many organs—including the liver and other splanchnic organs, skeletal muscle, bone, and others—contain small but variable quantities of nonpolar lipids [10]. Fat deposition in some of these locations is influenced by lifestyle and disease factors; in this regard, skeletal muscle and liver are noteworthy due to the association of increased fat deposition at these sites and adverse metabolic consequences, such as insulin resistance [11].

As with the previous comparison, the distinctions between FFM and skeletal muscle mass are noteworthy. It is frequently stated that FFM includes not only skeletal muscle but other components, such as organs, bone, and fluids. While well intentioned, such a statement intermeshes entities from multiple levels of assessment, namely the molecular (FFM) and organ/tissue (skeletal muscle, organs, bone). Molecularly, FFM consists of all nonfat molecules in the body, regardless of where they occur. The primary molecular categories within FFM include water (∼74%), protein (∼19%), mineral (∼6.5%), and a small residual component [10, 12]. While not discussed here, FFM may also be expected to include structural lipids (ie, polar lipids historically termed “*essential fat*” [13]). In contrast, skeletal muscle mass is the cumulative mass of the hundreds of anatomically defined skeletal muscles within the body. All major categories of molecules, including water, protein, minerals, glycogen, and lipids, are included within skeletal muscle tissue. While nearly all of skeletal muscle mass would be viewed as “lean” when considered at the molecular level (see Fig. 2), approximately 2% to 5% has been estimated to be lipid [10, 14], although the actual value likely varies based on the extent of intramuscular or intermuscular fat infiltration (eg, myosteatosis) [15]. Unlike the former comparison of FM and adipose tissue, the typical absolute quantities of FFM and skeletal muscle mass are notably disparate. Based on reference data, the quantity of FFM is more than double that of skeletal muscle mass (56.5 kg FFM vs 28 kg skeletal muscle mass for reference man; 42.0 kg FFM vs 17.0 kg skeletal muscle mass for reference woman [10]), although lifestyle practices such as exercise training could influence this relationship. In addition to the conceptual differences, the size disparity between FFM and skeletal muscle mass highlights the importance of avoiding a direct conflation of these entities.

While molecular- and organ/tissue-level entities are conceptually distinct, the relationship between them can be leveraged to bridge from more accessible measurement techniques to outcomes of interest at another assessment level. A notable example is the use of appendicular lean soft tissue from dual-energy x-ray absorptiometry (DXA) to estimate skeletal muscle mass using magnetic resonance imaging (MRI)-based equations [16-18]. While lean soft tissue in the trunk region contains a substantial contribution from organs, the lean soft tissue estimates of the appendages are subject to much less confounding when establishing a relationship to skeletal muscle mass. That is, the proportion of molecular level lean soft tissue in the appendages that overlaps with skeletal muscle tissue is notably higher than for the trunk or total body [16]. As such, strong relationships between appendicular lean soft tissue and whole-body or segmental skeletal muscle have been leveraged to estimate an organ/tissue component from available molecular-level data [19].

## Loss of Fat-Free Mass and Skeletal Muscle Mass During Weight Loss

**Figure 3.**
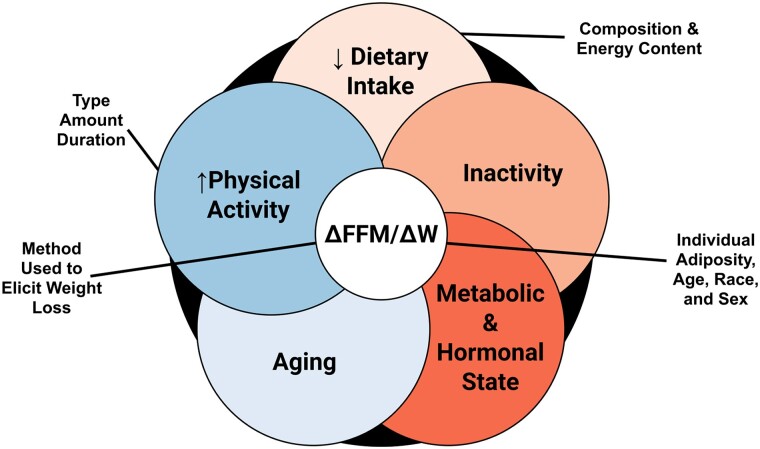
Potential contributors to the proportion of weight lost as fat-free mass. Adapted with permission from Heymsfield et al [20]. ΔFFM/ΔW, change in fat-free mass relative to the change in weight.

Historically, the “quarter FFM” rule stated that approximately 25% of weight can be expected to be lost as FFM. While there is some group-level support for this approximation, many factors can influence individual changes in FFM relative to weight loss—including dietary intake, physical activity, aging, the metabolic and hormonal state—and individual factors like adiposity, race, and sex [20] (Fig. 3). Given the current conversations about GLP-1RA treatments, the consideration of the method used to achieve weight loss is also noteworthy. A systematic review by Chaston et al [21] examined this question in the context of dietary, behavioral, pharmaceutical, and surgical interventions to produce substantial weight loss, albeit prior to widespread use of GLP-1RA. The median percentage of weight lost as FFM was 14% for low-calorie diets and approximately 23% for very low-calorie diets, regardless of whether exercise was included. Possible sex differences were also noted: When pooling estimates across dietary and behavioral weight loss interventions, the mean FFM loss was 27% for males and 20% for females. Limited evidence was available for pharmaceutical interventions (low-calorie diet plus sibutramine), with 2 included studies reporting FFM loss of more than 30% of weight loss. For surgical interventions, the median loss of FFM ranged from 18% to 31% across specific operations. While collectively supporting the “quarter FFM” rule as a general group-level approximation, direct comparisons within contemporary GLP-1RA trials are needed to establish the similarity of FFM losses across treatment types [22, 23].

**Figure 4.**
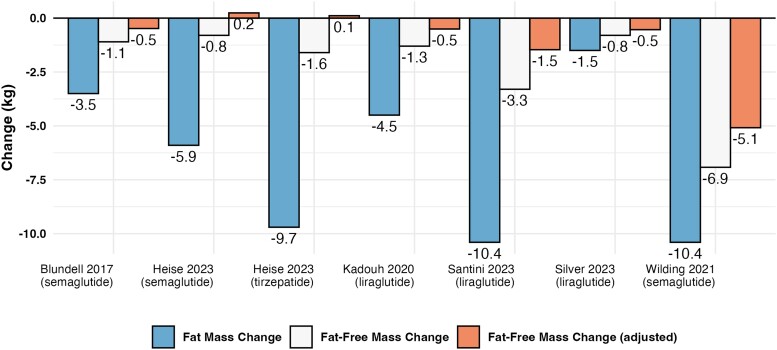
Adjustment of fat-free mass changes for obligatory loss of the fat-free component of adipose tissue. The fat-free component of adipose tissue (FFAT) is approximated from fat mass changes reported by contemporary glucagon-like peptide-1 receptor agonist (GLP-1RA) trials as: $FFAT\approx \frac{Fat\;mass}{0.85}\times 0.15$, when assuming that all fat mass loss is derived from adipose tissue and the fat content of adipose tissue is 85% [26, 27]. The fat-free component of adipose tissue is then subtracted from the reported fat-free mass changes, due to its obligatory nature, to provide an adjusted estimate of fat-free mass loss from non-adipose tissues. Data were obtained from published GLP-1RA trials [31-36].

As highlighted (see Fig. 2), adipose tissue has a fat-free or “lean” component that is composed primarily of water and protein and estimated to typically represent approximately 15% to 20% of adipose tissue mass [10]. When weight loss occurs, adipose tissue mass is typically reduced, leading to the loss of molecular level fat [24, 25] as well some degree of obligatory loss of the fat-free component of adipose tissue. When large magnitudes of weight loss occur, such as are common with the use of GLP-1RA drugs, the magnitude of obligatory FFM loss from adipose tissue may be sufficient to inappropriately influence the interpretation of total FFM loss, or the proportion of weight loss as FFM. However, the obligatory FFM loss from adipose tissue can be approximated mathematically [26, 27]. This adjustment leads to reductions in stated FFM loss in proportion with the magnitude of FM loss. For example, Abe et al [27] demonstrated how these corrections changed an apparent loss of FFM, relative to body mass lost, of 12% for weight loss in combination with resistance exercise to an increase of 4% [27, 28]. However, it is essential to note that such corrections assume that all FM loss is directly from adipose tissue. While this assumption is not entirely accurate [29, 30] and the previously described distinctions between molecular and organ/tissue components are important to recall, the assumption that the vast majority of FM loss occurs from adipose tissue is reasonable. As such, the theoretical calculations can be a useful heuristic for demonstrating obligatory loss from the fat-free component of adipose tissue. The same calculations can be applied to contemporary GLP1-RA weight loss trials, as demonstrated in Fig. 4.

**Figure 5.**
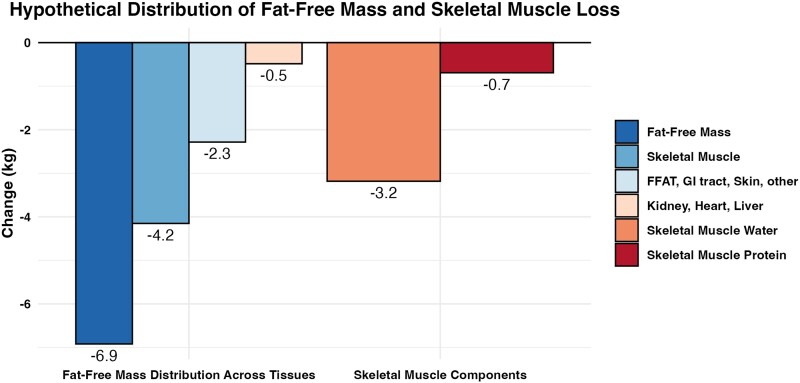
Hypothetical distribution of fat-free mass and skeletal muscle loss during weight loss. Relationships between total fat-free mass loss and loss of fat-free mass from specific tissues and organs presented by Bosy-Westphal et al [29] are hypothetically applied to fat-free mass loss with glucagon-like peptide-1 receptor agonist (GLP-1RA) treatment reported by Wilding et al [34]. While the proportions between fat-free mass loss may vary between weight loss methods, the data are illustrative of the multiple tissue or organ sources contributing to fat-free mass loss.

In addition to the consideration of the obligatory loss of FFM from adipose tissue, it is essential to recall that even corrected FFM is not directly synonymous with skeletal muscle mass. In many investigations, losses of FFM have been conflated with losses of skeletal muscle mass. However, research using MRI has demonstrated the varied tissue or organ sources of FFM loss during weight loss. Bosy-Westphal et al [29] found that in a group of women with overweight and obesity, a 9.5-kg loss of body mass resulted in a 1.5-kg loss of FFM during a low-energy diet. When examining the organ and tissue sources contributing to this molecular level loss of FFM, it was estimated that 0.9 kg (60% of FFM loss) originated from skeletal muscle, while 0.1 kg (7% of FFM loss) came from the kidney, heart, and liver; and 0.5 kg (33% of FFM loss) was due to the fat-free component of adipose tissue, the gastrointestinal tract, skin, or other unmeasured components. It is noteworthy that the estimation of the loss of FFM from adipose tissue would have been 1.4 kg using the previously discussed method (ie, $\frac{8.0\;kg\;FM\;loss}{0.85}\times 0.15=1.4\;kg$) [26, 27], while estimated change based on MRI quantification was smaller. Bosy-Westphal et al [29] further examined the skeletal muscle components being lost and estimated that, of the 0.9 kg of total skeletal muscle loss, 0.7 kg (46% of FFM loss) was due to reductions of water while only 0.15 kg (10% of FFM loss) was due to actual protein loss. While the proportions of different tissues lost may vary with distinct weight loss methods, the proportions reported by Bosy-Westphal et al [29] can be hypothetically applied to FFM loss reported in GLP-1RA trials for illustrative purposes (Fig. 5). While theoretical, these data nonetheless demonstrate that the entirety of FFM loss is not directly attributable to skeletal muscle and should not be interpreted as such. While the purpose of highlighting these considerations is not to minimize the importance of skeletal muscle for physiological function and well-being, they indicate that necessary context and nuance are warranted when interpreting FFM changes, particularly in the context of substantial weight loss.

## Minimizing Loss of Fat-Free Mass and Skeletal Muscle Mass

While appropriate interpretation of body composition changes and FFM loss is warranted, it is nonetheless a worthy goal to minimize the loss of FFM and skeletal muscle during weight loss, particularly due to the metabolic and functional importance of skeletal muscle [4, 5]. Two countermeasures worthy of consideration are exercise training and increased protein intake.

### Exercise Training

Exercise of varying modalities may notably mitigate FFM loss during weight loss. One systematic review found the percentage of energy restriction interventions yielding 15% or more of weight loss as FFM was 81% for energy restriction alone, compared to only 39% for energy restriction plus exercise [37]. While the majority of exercise interventions in this analysis were endurance training, several included resistance training or concurrent (ie, endurance plus resistance) training. In addition to FFM preservation, research specifically examining skeletal muscle mass, as estimated by MRI or computed tomography, has also supported a protective effect both of endurance and resistance exercise during energy restriction [28, 38-40]. For example, Ross et al [38] evaluated MRI appendicular skeletal muscle mass changes in women with obesity who followed an energy-restricted diet (1000 kcal/d below weight-maintenance needs) alone or in combination with endurance exercise. After the 16-week treatment period, similar weight reduction of approximately 11% was experienced by both groups; however, loss of appendicular skeletal muscle was observed in the diet-only group, but not in the group who additionally performed endurance exercise. Additionally, the loss of whole-body lean volume, estimated by MRI, averaged 14% in the diet-only group, but was only 2% in the group performing exercise. In another investigation, the same researchers similarly observed preservation of MRI skeletal muscle and lean volume when a 1000 kcal/d energy deficit was implemented in conjunction with resistance exercise in women with obesity [39]. Furthermore, a study directly comparing exercise modalities during a weight management program eliciting approximately 9% weight loss reported that the loss of thigh skeletal muscle volume was attenuated with resistance and concurrent training as compared to endurance training [28]. Collectively, these results suggest that exercise training mitigates skeletal muscle loss during weight loss, and programs including resistance training may be particularly effective [41].

In the context of GLP-1RA treatment, Lundgren et al [23] demonstrated body composition benefits of participation in an exercise program in adults being treated with liraglutide. During an initial 8-week low-calorie diet completed by all participants, FM was reduced by 7.3 kg, on average, while FFM was reduced by 5.1 kg. Subsequently, participants were randomly assigned to liraglutide, exercise, liraglutide + exercise, or placebo for 1 year. The exercise program consisted of 2 weekly group exercise sessions (including interval-based cycling and circuit training) and 2 weekly individual sessions (primarily cycling, running, or brisk walking). Relative to the point of randomization, only the liraglutide + exercise group experienced statistically significant weight loss, produced by a decrease in FM (−4.7 kg) without change in FFM (+0.5 kg). In contrast, the liraglutide group significantly decreased FM (−2.0 kg) with no change in FFM (0.0 kg), while the exercise group experienced no statistically significant decrease in FM (−1.4 kg) but increased FFM (+2.1 kg). The placebo group increased both FM and FFM in similar quantities (+2.6 and +2.9 kg, respectively). In summary, the addition of exercise to liraglutide potentiated fat loss with FFM preservation, while exercise alone increased FFM without significant fat loss. Interestingly, a subsequent report including a 1-year posttreatment period following the initial trial concluded that the treatments including exercise led to superior maintenance of body weight and composition 1 year after termination of treatment as compared to liraglutide alone [22]. It was also reported that all groups had similar increases in FFM from initial randomization to the end of the 1-year posttreatment period (ie, weeks 0-104). Additional research including skeletal muscle estimation may aid the interpretation of these FFM findings, and GLP-1RA trials including exercise throughout the entire weight loss intervention are warranted.

While many exercise modalities may offer health or body composition benefits [42], resistance training is considered the most effective nonpharmacological method of stimulating skeletal muscle growth or combatting muscle loss [43], with benefits observed not only for muscle mass, but also strength and physical function [44]. Although prolonged energy deficits employed during weight loss can attenuate resistance training-induced increases in FFM [45], resistance training can nonetheless mitigate losses of FFM during weight loss [28, 46]. Mechanistic research has demonstrated the ability of resistance training to prevent the typical reductions in both daily myofibrillar protein synthesis and postabsorptive muscle protein synthesis typically observed with energy restriction [47]. However, there is currently a lack of research examining the effects of resistance training alongside GLP-1RA administration, and this combined treatment approach should be investigated to determine if it enhances body composition changes, as well as supports superior body composition maintenance following treatment cessation. Interestingly, some preliminary work has supported possible benefits of GLP-1 and GLP-1RA for combatting muscle atrophy and myopathies related to inflammation. The putative effects—which include upregulation of myogenic factors, downregulation of atrophic factors, reductions in expression of inflammatory cytokines, mitochondrial preservation and biogenesis, and improvements in muscle microvasculature [48]—exhibit some overlap with responses to acute resistance exercise and adaptations to chronic resistance training [49-51]. Future work may clarify the relevance of these mechanisms in the context of combined GLP-1RA administration and resistance training.

Appropriately designed resistance training is generally viewed as the most efficient method of increasing skeletal muscle mass and strength in adults [52-54] and therefore represents an appropriate modality to promote skeletal muscle preservation during weight loss. The *Physical Activity Guidelines for Americans* [55], endorsed by the Centers for Disease Control and Prevention and American College of Sports Medicine, includes, in addition to 150 minutes/week of moderate-intensity aerobic (endurance) activity, a recommendation to perform muscle-strengthening activities of moderate or greater intensity that activate all major muscle groups on 2 or more days each week. While these recommendations promote resistance training, they are generic and lack prescriptive detail regarding optimal program design. In that regard, a separate position statement by the American College of Sports Medicine provides recommendations for resistance training to promote muscle size and strength in healthy adults with limited or no prior resistance training experience [53]. Major components of these recommendations are summarized in Table 2. Forthcoming clinical trials should examine the potential of varying modalities and “doses” of exercise to help attenuate losses of FFM and skeletal muscle mass during GLP-1RA treatment.

**Table 2.** Resistance training recommendations*^a^*

Variable	Recommendations
Exercise selection	Include a variety of exercises Target all major muscle groups*^b^* Use multiple muscle actions (concentric, eccentric, isometric) and bilateral and unilateral movements Perform multi-joint and single-joint exercises Use machines and/or free weights
Exercise frequency	2-3 d per wk
Exercise order	Larger before smaller muscle groups Multi-joint before single-joint Higher intensity before lower intensity
Load and repetitions	Highest weight that can safely be used for 8-12 repetitions per set Progression: increase weight when needed to maintain challenging stimulus in target repetition range
Sets	1-3 sets per exercise
Speed	Moderate velocity (not purposefully fast or slow)
Rest periods	1-2 min between sets

^*a*^Based on selected recommendations from the American College of Sports Medicine Position Statement: Progression Models in Resistance Training for Healthy Adults [53].

^*b*^Legs (quadriceps, hamstrings, gluteals, etc), back, chest, shoulders, arms, core.

### Protein Intake

In addition to exercise, select dietary interventions may help maintain skeletal muscle mass during weight loss. One strategy in particular that has demonstrated potential is increased dietary protein intake. In the United States, the recommended dietary allowance (RDA) for protein is set at 0.8 g/kg for most adults, while the acceptable macronutrient distribution range is 10% to 35% of total energy. Multiple lines of research support the contention that consuming protein higher than the RDA offers an FFM or skeletal muscle preservation benefit [56]. For example, it has been demonstrated that consuming twice the RDA (1.6 g/kg) of protein during a 40% energy deficit reduces FFM loss [57]. Other research found that intakes of 1.1 to 1.6 g/kg preserved FFM during weight loss better than 0.6 to 0.9 g/kg in individuals with overweight and obesity [58, 59]. Additionally, a meta-analysis of weight loss studies conducted in adults with mean ages of 50 years or older concluded that FFM retention was improved by consumption of higher protein diets (≥25% of energy intake or >1.0 g/kg), although the magnitude of this benefit was modest (0.45-0.83 kg) [60]. It has also been observed that protein supplementation (21 g protein, including ∼11 g essential amino acids and ∼3 g leucine) led to an increase in estimated appendicular skeletal muscle mass during a 13-week weight loss plus resistance training program in older adults with obesity, as compared to an isocaloric placebo [61]. In this case, supplementation resulted in a total daily protein intake of 1.1 g/kg as compared to 0.85 g/kg in the placebo group. While there is limited research to inform differential protein requirements with GLP-1RA treatment, emphasizing protein intake for patients using GLP-1RA may be relevant, as some evidence indicates an attenuation of the increase in protein intake, relative to total energy intake, with GLP-1RA therapies as compared to standard caloric restriction [31]. However, additional research confirming the influence of GLP-1RA on nutrient intake and examining the potential for manipulation of protein intake to promote FFM and skeletal muscle preservation is warranted.

Collectively, a protein intake of 1.2 g/kg or greater or 20% or greater of total energy may be an appropriate target during weight loss. However, the implications of prescribing protein intake based on current body mass in individuals with obesity should be considered, with an alternative being to base calculations on target body mass if intake based on current body mass is not readily achievable. Practical strategies for promoting a higher protein intake include targeting 20 to 40 g of protein at each eating occasion, a quantity consistent with maximal stimulation of muscle protein synthesis [62]; consuming protein 3 or more separate times each day; and implementing behaviors that promote the target daily protein intake without the need for continual nutrient tracking, such as planning each meal around a high-quality protein source and having high-protein foods readily available. In addition to FFM and skeletal muscle preservation, higher protein intakes have additional potential benefits for appetite regulation, improving body composition, and influencing other health components [59], which could play a supportive role in GLP-1RA–induced weight loss.

## Conclusions

Widespread use of GLP-1RA drugs in research and clinical practice underscores the importance of appropriate interpretation of body composition changes during weight loss. Questions regarding FFM and skeletal muscle loss with varying weight loss treatments can be aided by a recollection of fundamental body composition principles, such as the distinction between molecular-level and organ/tissue-level components. In this regard, changes in FFM should not be directly conflated with changes in skeletal muscle mass. Additionally, the potential for obligatory loss of FFM from adipose tissue should be considered when interpreting FFM changes with large magnitudes of weight loss, such as those frequently achieved with GLP-1RA treatment. While these conceptual frameworks can aid researchers and clinicians in accurately evaluating and contextualizing body composition changes in patient populations, skeletal muscle preservation during weight loss is still a worthy goal. In this regard, exercise, particularly resistance training, and increased dietary protein intake are two countermeasures that may synergistically promote FFM and skeletal muscle retention during weight loss. The potential for these practices to enhance body composition outcomes achieved by GLP-1RA therapies should be the focus of future clinical research.

## Data Availability

N/A.

## Abbreviations

3DO: 3-dimensional optical imaging
ADP: air displacement plethysmography
BIA: bioelectrical impedance analysis
BIS: bioimpedance spectroscopy
CT: computed tomography
DXA: dual-energy x-ray absorptiometry
FFM: fat-free mass
FM: fat mass
GLP-1RA: glucagon-like peptide-1 receptor agonist
MRI: magnetic resonance imaging
RDA: recommended dietary allowance
SKF: skinfold thickness assessments
US: ultrasonography
UWW: underwater weighing
